# The impact of multidisciplinary team management on outcome of hepatic resection in liver-limited colorectal metastases

**DOI:** 10.1038/s41598-020-67676-1

**Published:** 2020-07-02

**Authors:** Michele Basso, Salvatore Corallo, Maria Alessandra Calegari, Ina Valeria Zurlo, Francesco Ardito, Maria Vellone, Silvio Marchesani, Armando Orlandi, Vincenzo Dadduzio, Giovanni Fucà, Carmela Di Dio, Caterina Mele, Brunella Barbaro, Antonia Strippoli, Alessandro Coppola, Alessandra Cassano, Emilio Bria, Carlo Antonio Barone, Felice Giuliante

**Affiliations:** 1 Oncologia Medica, Fondazione Policlinico Universitario “A. Gemelli” – IRCCS, Largo Francesco Vito 1, 00168 Rome, Italy; 2 Oncologia Medica, Fondazione IRCCS Istituto Nazionale dei Tumori, Milan, Italy; 3 Oncologia Medica, Università Cattolica del Sacro Cuore-IRCCS, Rome, Italy; 4 Chirurgia Epatobiliare, Università Cattolica del Sacro Cuore-IRCCS, Rome, Italy; 5 Oncologia Medica 1, Istituto Oncologico Veneto IRCCS, Padua, Italy; 6 Chirurgia Epatobiliare, Fondazione Policlinico Universitario “A. Gemelli” – IRCCS, Rome, Italy; 7 Radiologia Diagnostica ed Interventistica Generale, Università Cattolica del Sacro Cuore – IRCCS, Rome, Italy

**Keywords:** Oncology, Gastrointestinal cancer

## Abstract

Hepatic resection is the gold standard treatment for patients affected by liver-limited colorectal metastases. Reports addressing the impact of multidisciplinary team (MDT) evaluation on survival are controversial. The aim of this study was to evaluate the benefit of MDT management in these patients in our Institution experience. The objective of the analysis was to compare survivals of patients managed within our MDT (MDT cohort) to those of patients referred to surgery from other hospitals without MDT discussion (non-MDT cohort). Of the 523 patients, 229 were included in the MDT cohort and 294 in the non-MDT cohort. No difference between the two groups was found in terms of median overall survival (52.5 vs 53.6 months; HR 1.13; 95% CI, 0.88–1.45; *p* = 0.344). In the MDT cohort there was a higher number of metastases (4.5 vs 2.7; *p* < 0.0001). The median duration of chemotherapy was lower in MDT patients (8 vs 10 cycles; *p* < 0.001). Post-operative morbidity was lower in the MDT cohort (6.2 vs 21.5%; *p* < 0.001). One hundred and ninety-seven patients in each group were matched by propensity score and no significant difference was observed between the two groups in terms of OS and DFS. Our study does not demonstrate a survival benefit from MDT management, but it allows surgery to patients with a more advanced disease. MDT assessment reduces the median duration of chemotherapy and post-operative morbidities.

## Introduction

Colorectal cancer (CRC) is the third most commonly diagnosed cancer in developed countries^1^, ranking second in frequency in Europe^2^, and, despite recent improvements in diagnosis and treatment, is still the third leading cause of cancer-related death. About 25% of CRC patients present at diagnosis with liver metastases and up to half will develop liver metastases over the course of their disease. The introduction of modern combination chemotherapy regimens and targeted therapies^3^ and the recent advances in surgical techniques^4^ have dramatically improved the survival of patients with liver-limited colorectal metastases over the last decade^5^. Nowadays, hepatic resection is the gold standard treatment for patients with liver-limited colorectal metastases with 5- and 10-years survival rates reaching up to 60% and 20%, respectively^6,7^. The use of liver remodelling strategies (including portal vein ligation or embolization) and effective conversion treatments with chemotherapeutic (irinotecan and oxaliplatin) and targeted (bevacizumab, cetuximab and panitumumab) agents has contributed to increase to about 25% the rate of patients suitable for surgery^8^.

In order to further select those patients who may benefit from liver resection, a multidisciplinary team (MDT) approach, including key figures such as medical oncologists, surgeons and radiologists, has been strongly recommended. Through a deep interaction of different specialties, a MDT evaluation could also ensure that all suitable patients are referred to liver resection and provide patients with the most adequate and tailored management^9,10^.

Despite being largely accepted that MDT management might guarantee a more accurate assessment and referral of patients, reports discussing the impact of MDTs on patients’ outcomes are controversial^11,12^ and up to now there are no strong evidences for routinely MDT discussion^13–15^. The aim of this study was to evaluate the benefit of MDT management in patients with liver-limited colorectal metastases in our single institution experience, comparing outcomes of patients managed within MDT to those referred to surgery without MDT discussion.

## Patients and methods

A prospective database was established in the Hepatobiliary Surgery Unit (HbSU) at Fondazione Policlinico Universitario “A. Gemelli”—IRCCS in Rome in January 1987 for all consecutive admissions related to possible liver resection. This database contains information prospectively collected by the HbSU and by the Oncologic Unit (OU) of our Center on patients who underwent liver resection for colorectal liver metastases after a MDT discussion. Moreover, the above-mentioned database contains also data on liver resections performed on patients referred to our HbSU from other hospitals without a MDT discussion. Information collected includes demographic data, pathological and molecular features, surgical details, treatments regimens and follow-up reports until recurrence and/or death. Information has been periodically updated. Given the observational, non-interventional and retrospective nature, the study had no influence on the patient’s course of treatments. All patients data were collected anonymously. All patients signed an informed consent for chemotherapy and/or surgery, including consent for data collection and tissue sample use. The study was conducted in accordance with the Declaration of Helsinky and was approved by the Ethic Commettee of Fondazione Policlinico Universitario “A Gemelli”-IRCCS.

Records from January 2006 to December 2016 were retrieved from the database and analyzed. Pre-operative disease assessment was determined on computerized tomography (CT) scan of the chest, abdomen and pelvis, magnetic resonance imaging (MRI) of the liver and/or positron-emission-tomography (PET-CT), depending on clinical needs. In our policy there were no predefined criteria of unresectability with regard to number, size, and bilaterality of colorectal liver-limited metastases (CRLM). Lesions were defined as resectable when all disease could be removed with negative margins, leaving an adequate liver remnant. Unresectability was defined as technical unresectability because of inadequate liver remnant or the inability to remove all CRLM either by a 1- or 2-stage procedure. An anticipated risk of R1 resection was not a contraindication to liver resection, although our preferred policy has always been to obtain a tumor-free margin of 1 cm or more whenever possible^16^. Peri-operative mortality (defined as 90-day mortality) and morbidity (complications were scored according to the Clavien grading system^17^) were recorded. Information about post-operative therapy and further surgical procedures was prospectively collected during periodic follow-up visits and recorded in the database. The time and the site of recurrence was established by means of clinical imaging (CT scans, abdominal MRIs or PET-CT scans) during follow-up period.

### Statistical analysis

This is a retrospective observational study. The objective of the analysis was to compare survivals of patients managed within the MDT of Fondazione Policlinico “A. Gemelli”—IRCCS (MDT cohort) to those of patients referred to surgery from other hospitals without MDT discussion (non-MDT cohort). Although there are no conclusive data in literature, we suppose a 15% benefit in terms of overall survival for the MDT cohort versus the non-MDT one. Hyphothesizing an α-error of 0.05 and a β-error of 0.1, 222 patients in each group will give the study a 90% power to detect the supposed difference.

Primary endpoints were disease free survival (DFS) and overall survival (OS). DFS was calculated as the time from the date of complete liver resection to the date of first evidence of recurrence of disease during long-term follow-up. OS was calculated as the time from the date of liver resection to the date of death or to the last follow-up visit (censored data). Differences in baseline characteristics and in post-operative morbidity were also evaluated.

Continous and categorical clinical variables were presented as median (inter-quartile range: 25–75%), mean and number of patients (proportion) and compared using chi-squared tests, whereas numerical variables were examined using Student’s *t* tests. Survival curves were plotted using the Kaplan–Meier methods and compared using the log-rank tests.

To assess putative bias of patient characteristics among the two groups, a propensity score analysis as a more refined statistical method to adjust for potential baseline confounding variables was performed. Propensity score was calculated with a multivariable logistic regression model including the following prognostic variables: age, timing of metastases, number of metastases, larger diameter of metastases, systemic treatments before surgery, location of metastases (monolobar or bilobar). The survival analysis was adjusted using the Cox’s proportional hazard model including treatment group (MDT or non-MDT) and propensity score for all patients. A 1-to-1 matching was performed using the propensity score (propensity score-matched data-set) and patients in the two cohorts were matched by a difference of propensity score within 0.5.

Tests were considered statistically significant with a *p* value of less than 0.05. Statistical analysis was performed using R software (version 3.5.0).

## Results

### Patients characteristics, MDT cohort

**Figure 1:**
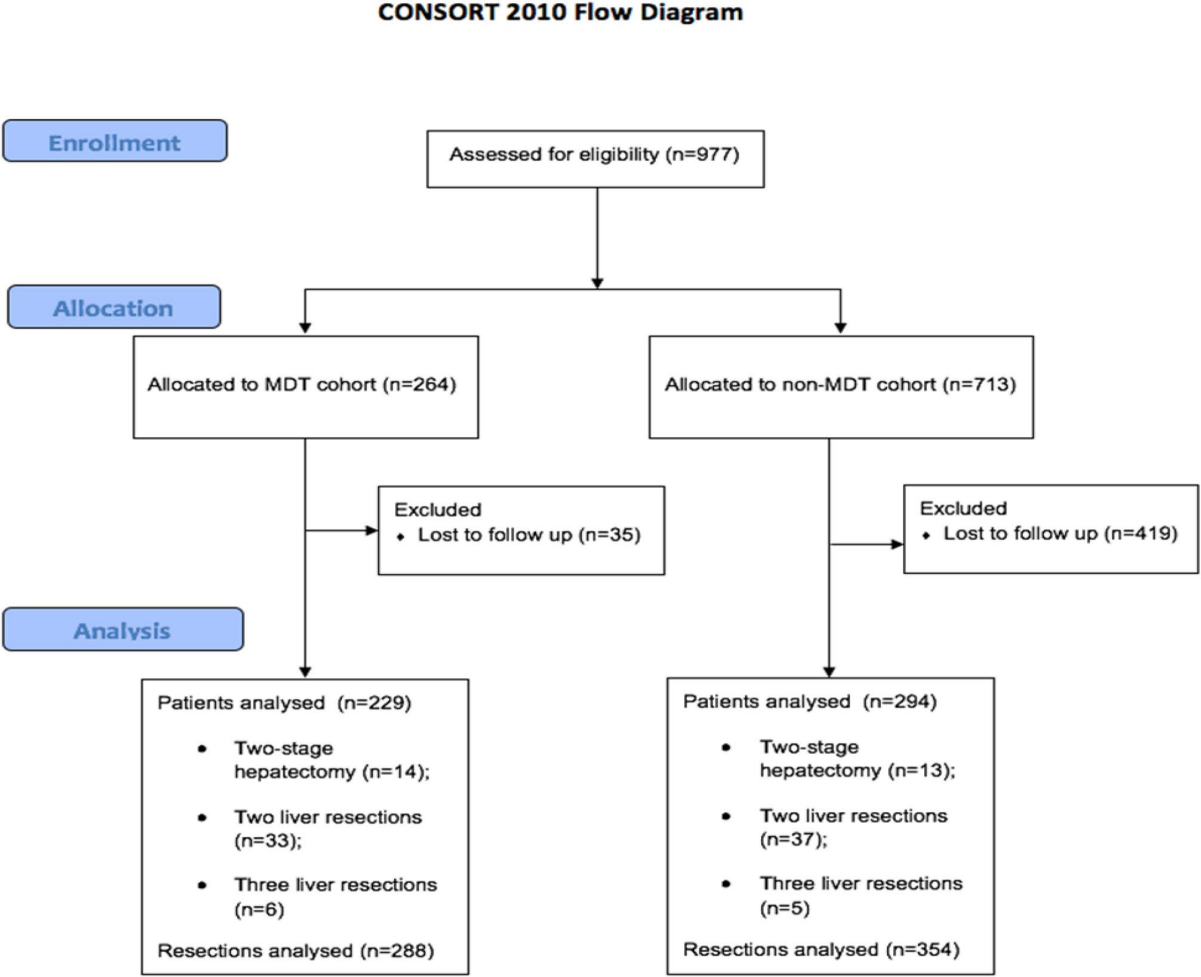
CONSORT diagram.

From January 2006 to December 2016 a total of 264 patients with liver-limited colorectal metastases underwent liver resection following a MDT discussion. Of those, 35 patients were excluded due to the lack of adequate follow up (Fig. 1). Of 229 patients included in the analysis, 14 patients (6.1%) received a two-stage hepatectomy for bilobar liver metastases, 33 patients (14.4%) underwent to a second hepatic resection following local recurrence of disease and 6 patients (2.6%) had three liver resections due to multiple recurrences of disease, accounting for 288 liver resections following a MDT discussion over the study period. Patients’ details are outlined in Table 1.

**Table 1:** Patients’ characteristics.

Characteristics	MDT cohort, N = 229 (%)	Non-MDT cohort, N = 294 (%)	*p* value
**Gender**			
Male	142 (62)	171 (58.2)	0.373
Female	87 (38)	123 (41.8)	
**Age**			
Median years (IQR)	64 (57–71)	64 (56–70)	0.563
< 60 years	77 (33.6)	106 (36)	0.712
≥ 60 years	152 (66.4)	188 (64)	
**Onset of metastases**			
Synchronous	141 (61.6)	183 (62.2)	0.875
Metachronous	88 (38.4)	111 (37.8)	
**Number of metastases at diagnosis**			
Mean (95% CI)	3.9 (3.4–4.3)	2.8 (2.4–3.1)	< 0.001
Single metastasis	85 (37.1)	127 (43.2)	
2–3 metastases	59 (25.8)	99 (33.7)	< 0.015*
> 3 metastases	85 (37.1)	68 (23.1)	
**Size of metastases at diagnosis**			
Mean (95%CI)	4 (3.6–4.4)	3.7 (3.4–4.0)	0.170
≤ 3 cm	105 (45.9)	158 (53.7)	0.073
> 3 cm	124 (54.1)	136 (46.3)	
**Location of metastases**			
Monolobar	120 (52.4)	174 (59.2)	0.121
Bilobar	109 (47.6)	120 (40.8)	

*IQR* interquartile range, *95% CI* 95% confidence interval.

*Statistically significant.

Most patients were male (62.0%). Median age was 64 years (range 24–83) and 77 patients (33.6%) were younger than 60 years. Most patients had synchronous metastases (61.6%). Eighty-five patients (37.1%) had a single metastasis. One-hundred forty-four patients (62.9%) had ≤ 3 metastases, whereas 85 patients (37.1%) had > 3 metastases. Median tumor size was 4 cm (range 0.5–25 cm); 105 patients (45.9%) had a median metastases size ≤ 3 cm and 124 (54.1%) had a median metastases size > 3 cm. In 120 patients (52.4%) a monolobar involvement was documented, while 109 patients (47.6%) had bilobar metastases.

Among all 288 liver resections, 129 cases (44.8%) were considered resectable at diagnosis and liver resection was done as primary treatment, while 159 cases (55.2%) were considered initially unresectable and liver resection was performed after pre-operative chemotherapy. Median duration of pre-operative chemotherapy was 8 cycles (range 2–24). Treatment regimens are outlined in Table 2. After pre-operative chemotherapy, one case (0.62%) obtained a complete radiological response, 129 cases (81.13%) achieved a partial response, 19 cases (11.95%) experienced stable disease and 10 cases (6.3%) had progressive disease. In patients receiving pre-operative chemotherapy a pathological complete response was achieved in nine cases. One-hundred fifty-nine cases (55%) received post-operative chemotherapy following liver resection: median duration of treatment was 6 cycles (range 2–12 cycles). Peri- or post-operative complications occurred in 18 cases (6.2%) but no patient died. Details concerning peri-operative morbidity are described in Table 3.

**Table 2:** Chemotherapy regimens and responses.

	MDT cohort, N = 229(%)	Non-MDT cohort, N = 294 (%)	*p* value
**No. of patients receiving pre-operative CT**	159 (69.4)	211 (71.7)	0.006
**Regimens**			
Oxaliplatin-containing regimens	79 (49.7)	103 (48.8)	0.118
Irinotecan-containing regimens	67 (42.1)	74 (35.1)	0.166
Oxaliplatin and irinotecan containing regimens (triplet)	10 (6.3)	31 (14.7)	0.011*
Monochemoterapy with fluoropyrimidines	3 (1.9)	3 (1.4)	0.726
**Biological agents**			
Anti-VEGF	53 (33.3)	70 (33.1)	0.980
Anti-EGFR	52 (32.7)	34 (16.1)	< 0.0001*
**Median number of cycles (range)**	8 (2–24)	10 (1–38)	0.002*
**Radiological response to chemotherapy**			
Complete response (CR)	1 (0.6)	0	0.257
Partial response (PR)	129 (81.1)	128 (60.7)	0.004*
Stable disease (SD)	19 (12)	32 (15.2)	0.322
Progressive disease (PD)	10 (6.3)	51 (24.1)	< 0.0001*

*CT* chemotherapy.

*Statistically significant.

**Table 3:** Post-operative mordidities.

	MDT cohort, n (%)	Non-MDT cohort, n (%)	*p* value
**Liver resections**	288	354	
**Morbidities**	18 (6.2%)	76 (21.5%)	< 0.0001*
Liver abscess	6 (33.4)	14 (18.4)	0.164
Abdominal bleeding	1 (5.5)	5 (6.6)	0.873
Biliary fistula	1 (5.5)	12 (15.8)	0.258
Intestinal occlusion and/or perforation	3 (16.7)	4 (5.3)	0.097
Infection	3 (16.7)	14 (18.4)	0.862
Liver failure	3 (16.7)	8 (10.5)	0.466
Other (thrombosis, etc.)	1 (5.5)	19 (25)	0.070

*Statistically significant.

### Patients characteristics, non-MDT cohort

Between January 2006 and December 2016, a total of 713 patients were referred to our HbSU from other centers without a MDT discussion. Of those, 419 patients were lost to follow-up after liver resection and were excluded from our analysis (Fig. 1). Among the 294 patients with adequate follow-up information, 13 (4.4%) underwent to a two-stage hepatectomy for bilobar disease, 37 (12.5%) had a second hepatic resection due to liver recurrence and 5 (1.7%) had more than two liver resections due to multiple relapses. Patients’ details are outlined in Table 1.

Most patients were male (58.2%). Median age was 64 years (range 29–87) and 106 patients (36.0%) were younger than 60 years. Most patients had synchronous metastases (62.2%). One-hundred twenty-seven patients (43.2%) had a single metastasis. Two-hundred twenty-six patients (76.9%) had ≤ 3 metastases, whereas 68 patients (23.1%) had > 3 metastases. Median tumor size was 3.6 cm (range 0.1–18 cm); 158 patients (53.7%) had a median metastases size ≤ 3 cm and 136 (46.3%) had a median metastases size > 3 cm. In 174 patients (59.2%) a monolobar involvement was documented, while 120 patients (40.8%) had bilobar disease.

Among 354 liver resections performed, 211 (59.6%) were considered initially unresectable and received pre-operative chemotherapy before liver resection, while 143 cases (40.4%) were considered resectable at diagnosis. Treatment regimens are outlined in Table 2. After pre-operative chemotherapy, 128 cases (60.7%) had a partial response, 32 cases (15.2%) obtained stable disease and 51 cases (24.1%) experienced progressive disease. Median duration of pre-operative chemotherapy was 10 cycles (range 1–38). No adequate information about post-operative chemotherapy could be retrieved from the registry database for this group of patients. Peri- or post-operative complications occurred in 76 cases (21.5%) but post-operative mortality was nil. Data concerning peri-operative morbidity are reported in Table 3.

### Comparison between groups and survival analysis

Comparison of clinical characteristics of the two groups of patients showed similar median age, gender, site and onset of metastases and median tumor size but a marginally significant difference in the median size of metastases was found (< 3 cm: 45.9 vs 53.7%; > 3 cm: 54.1 vs 46.3%; *p* = 0.07) (Table 1). A clear statistically significant difference in the median number of liver metastases resulted comparing patients discussed in the MDT and those referred to our HbSU from other hospital without a MDT discussion (mean 3.9 vs 2.8: *p* = 0.001). The distribution of liver resections over the time-period analyzed was almost equal in the two study groups.

The percentage of patients receiving pre-operative chemotherapy was similar between the two groups but the mean duration of perioperative chemotherapy was significantly lower in MDT cohort patients than in non-MDT cohort patients (8 vs 10 cycles with a mean difference of 1.7 m, accounting for 2 cycles; 95% CI: 0.67–2.76; *p* = 0.002). No significant differences was found concerning the choice of chemotherapy schedules. Nevertheless, anti-EGFR agents were used more frequently in patients belonging to the MDT group (32.7 vs 16.1%; *p* < 0.001). In contrast, aggressive combination chemotherapy containing both irinotecan and oxaliplatin was more frequently used among patients referred from other hospitals (6.3 vs 14.7; *p* = 0.011). Despite this, response to chemotherapy was higher in patients belonging to the MDT group, with an objective response rate of 81.7% compared to 60.7% of the non-MDT cohort (*p* = 0.004).

Other important differences between the two groups were the percentage of patients undergoing surgery with evidence of disease progression (6.3 vs 24.1%; *p* < 0.0001) and the rate of post-operative morbidities (6.2 vs 21.5%; *p* < 0.001) which were both higher in non-MDT patients.

**Figure 2:**
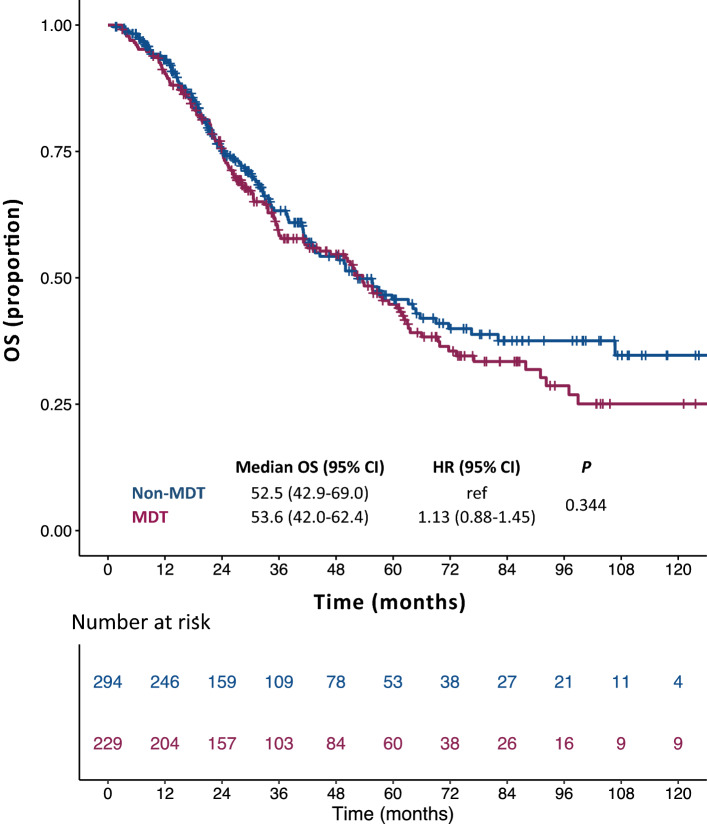
Kaplan–Meier curves for overall survival. *OS* overall survival, *non-MDT* non multidisciplinary team cohort, *MDT* multidisciplinary team cohort, *HR* hazard ratio; *CI* confidence interval.

**Figure 3:**
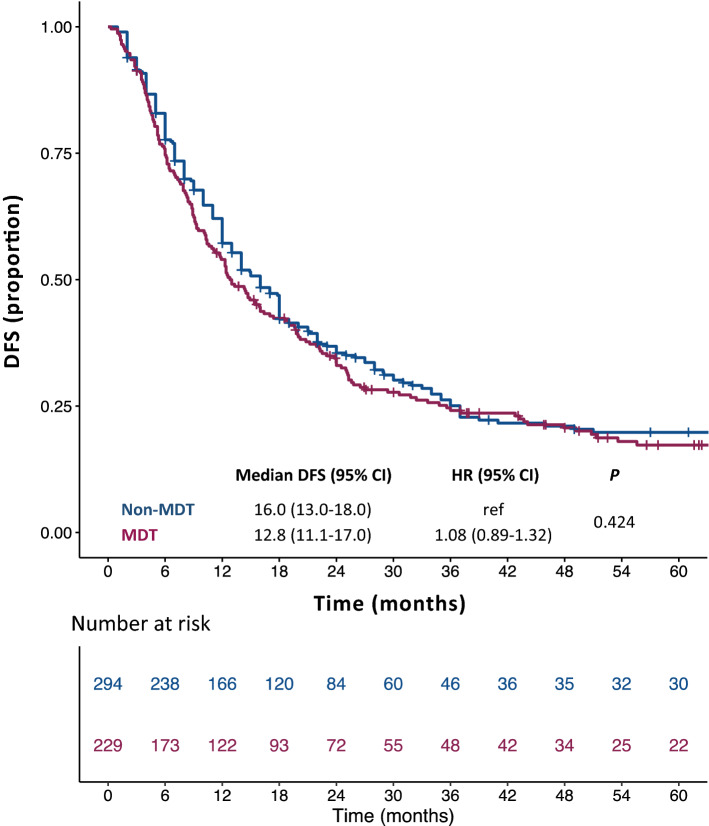
Kaplan–Meier curves for disease-free survival**.** *DFS* disease-free survival, *non-MDT* non multidisciplinary team cohort, *MDT* multidisciplinary team cohort, *HR* hazard ratio, *CI* confidence interval.

At a median follow-up of 56.6 months the median OS was 53.6 months in the MDT cohort (95% CI, 42.0–62.4; mean: 73.0 months) and 52.5 months in the non-MDT cohort (95% CI, 42.9–69.0; mean: 73.9 months). There was no significant OS difference between the two groups [unadjusted HR of non-MDT to MDT cohort: 1.13; 95% CI, 0.88–1.45; *p* = 0.344 (Fig. 2)]. No statistically significant difference was shown also in terms of DFS: median DFS was 12.8 months in the MDT cohort (95% CI, 11.1–17.0; mean: 41.8 months) and 16.0 months in the non-MDT cohort (95% CI, 13.0–18.0; mean: 40.4 months). The unadjusted HR of non-MDT to MDT cohort was 1.08 [95% CI, 089–1.32; *p* = 0.424 (Fig. 3)].

One hundred and ninety-seven patients in each group were matched by propensity score. No significant difference was observed between the two groups in terms of OS [adjusted HR 1.00; 95% CI: 0.68–1.47; *p* = 0.999, (Supplementary Figure 1)] nor DFS [HR 0.98; 95% CI: 0.73–1.33, *p* = 0.939; (Supplementary Figure 2)].

## Discussion

Although retrospective, this study is one of the largest on the clinical impact of a multidisciplinary management of patients affected by colorectal liver metastases in comparison with a non-structured referral to surgeons. The strength of this analysis relies on the largeness of the sample size and on the presence of a single surgical team, to whom all patients have been referred to for liver resection. Another important point is that the whole population, both in the MDT and in the non-MDT arm, was enrolled during the same time interval, thus minimizing the bias related to changes in treatment protocols, surgical procedures or surgeons’ expertise.

Our study did not demonstrate any statistically significant survival benefit from MDT management of patients with liver-limited colorectal metastases. Therefore, the hypothesis that multidisciplinary evaluation could improve the outcome of patients with liver-limited colorectal metastases has not been confirmed. A possible explanation for the absence of any significant survival difference between the two groups could derive from the centralization of surgery in the same center. Indeed, in the case of liver-limited colorectal metastases a main role is played by surgery expertise. Therefore the centralization of complex surgical procedures has extended the benefits of experience and ability of the same high volume center to the whole population of patients, both in the MDT and non-MDT arm. Furthermore, the availability in the last years of multiple lines of treatment in CRC allowed all patients to be exposed to effective post-progression therapies. Last, but not least, the very good overall survival of both arms may reflect a low-risk population, for which the benefit of MTD could be lower or more difficult to be demostrated. Indeed, a recent review by Look Hong et al.^18^, although not focused on colorectal liver metastases, analyzed 21 studies that described multidisciplinary cancer care and its relation to patient survival and concluded that to date it is not possible to assert a causal relationship between multidisciplinary care and patient survival.

Nevertheless, a deeper analysis of our data suggests that concluding that MDT management does not impact on survival of patients affected by colorectal liver metastases is simplistic. Indeed, a comparison of the two populations in the study reveals that patients belonging to the MDT group display worse prognostic features. These patients show a more widespread liver disease, due to higher number of metastases (> 3 metastases: 37.1 vs 23.1%), larger metastases (> 3 cm: 54.1 vs 46.3%) and bilateral involvement (48.0 vs 40.0%). Overall, these data clearly suggest a higher burden of disease for patients belonging to the MDT group.

Another important point of our study concerns the chemotherapy protocols used in peri-operative treatments. Patients included in the MDT group received combination regimens including an anti-EGFR agent more frequently compared to those belonging to the non-MDT group (32.7 vs 16.1%). On the other hand, patients belonging to the non-MDT group, although harboring a minor disease burden, received more frequently an aggressive triplet combination regimen (14.7 vs 6.3%). Moreover, patients belonging to the non-MDT group received a significantly higher number of chemotherapy cycles before surgery (10 vs 8; *p* = 0.002). Taken together, these differences offer a view on the role of the MDT and its choices, but it could also explain the higher rate of post-operative morbidities (6.2 vs 21.5%; *p* < 0.001) observed in the non-MDT group. Moreover, the fact that non-MDT patients underwent surgery with evidence of disease progression (a well-recognized negative prognostic factor) more frequently compared to MDT patients (24.3 vs 6.3%) suggests a less organized clinical pathway.

In conclusion, although our study does not demonstrate an overall survival benefit in favor of MDT management of patients with liver-limited mCRC, our analysis shows that patients managed within a MDT setting receive a lower number of chemotherapy cycles, anticipate surgery and experience a significantly lower rate of post-operative morbidities. Moreover, our study suggests that MDT management allows conversion to surgery of patients with a more widespread liver disease. This study was not aimed to compare the percentage of patients converted to surgery among those discussed in a MDT setting or directly referred to surgery without a MDT discussion. Nevertheless, given our results, it is conceivable that the rate of patients converted to surgery with curative intent might be higher in the MDT setting when compared to patients not managed in a MDT setting. Whether such inference lined up with reality it would represent the most important effect of MDT discussion. Unfortunately, it is very difficult to confirm such hypothesis in a prospective randomized study due to ethical and methodological concerns.
